# Longitudinal rotation: a new way to detect the cardiotoxicity of anthracycline-based chemotherapy in breast cancer patients

**DOI:** 10.18632/oncotarget.19585

**Published:** 2017-07-26

**Authors:** Jun Huang, Zi-Ning Yan, Yi-Fei Rui, Dan Shen, Li Fan, Dong-Liang Chen

**Affiliations:** ^1^ Department of Echocardiography, Changzhou No.2 People's Hospital Affiliated to NanJing Medical University, Changzhou 213003, China

**Keywords:** breast cancer, cardiotoxicity, chemotherapy, longitudinal rotation, anthracycline-based

## Abstract

**Background and aims:**

The study was to compare cardiac parameters before and after anthracycline-based chemotherapy and identify a parameter for detecting cardiotoxicity in breast cancer patients.

**Methods:**

Cardiac function in 43 female breast cancer patients was evaluated at three time points: baseline, 1-3 days before the initiation of anthracycline-based chemotherapy; 3 weeks and 6 months after the final cycle of chemotherapy. At each visit, the peak longitudinal velocity; strain rate; peak systolic strain; peak systolic longitudinal displacement, and segmental and global longitudinal rotation degrees of the left ventricular were measured.

**Results:**

The peak early-diastole left ventricular wall velocity at baseline was significantly higher than the values at 3 weeks and 6 months after the final cycle of chemotherapy. The absolute value of the lateral wall peak systolic longitudinal rotation degrees was significantly higher at baseline than at 3 weeks and 6 months after the final cycle of chemotherapy, whereas the absolute value of the global peak systolic longitudinal rotation degrees at baseline was significantly lower than the values at 3 weeks and 6 months after the final cycle of chemotherapy. None of the measured parameters differed significantly between the 3 weeks and 6 months after the final cycle of chemotherapy.

**Conclusions:**

Cardiac diastolic and systolic dysfunction was found after anthracycline-based chemotherapy in this study, and the peak systolic longitudinal rotation degrees can be used to detect dysfunction after chemotherapy. The cardiotoxicity of epirubicin-based chemotherapy is stronger than that of therarubicin-based chemotherapy.

## INTRODUCTION

Breast cancer is one of the most common malignant tumors, and now it is becoming a leading cause of cancer-related death in women worldwide [1–2]. The treatment for breast cancer involves a combination of surgery, chemotherapy, and radiotherapy [3]. Over the past decades, the mortality rate among breast cancer patients has decreased. However, cardiotoxicity induced by the chemotherapy has become a leading cause of morbidity and mortality in survivors [4–5]. To prevent heart failure caused by cancer therapy, consideration of potential cardiotoxicity is very important.

Epirubicin and therarubicin, as common anthracyclines, are widely used chemotherapy drugs for reducing the recurrence of breast cancer in China. However, the use of these drugs is limited by their cardiotoxicity. The cardiotoxicity of anthracycline-based chemotherapy may be associated with the cumulative dose, other associated cytotoxic drugs, or the advanced age of patients [6–7]. Previous studies found that the cardiotoxicity can appear during or after the chemotherapy in breast cancer patients [6, 8]. Left ventricular ejection fraction (LVEF) measurement by two-dimensional echocardiography is the most commonly used method for detecting cardiotoxicity, which is most commonly defined as a ≥5% or ≥10% reduction in the LVEF from baseline to an LVEF <55% in symptomatic patients and asymptomatic patients, respectively [9]. However, the use of LVEF has limitations for early detection of cardiotoxicity because it cannot reflect the early dysfunction of the heart [5].

With the development of various imaging techniques, such as tissue Doppler imaging (TDI), speckle tracking echocardiography (STE, two-dimensional or three-dimensional), and cardiac magnetic resonance imaging (CMRI), the cardiotoxicity of anthracycline-based chemotherapy can be detected with greater convenience. However, the angle dependency of TDI makes these results difficult to reproduce [10]. Although CMRI is considered the gold standard for evaluating LV volumes and EF, its lack of availability and high cost limit its routine use [11–12]. STE is an ideal method for detecting cardiotoxicity caused by chemotherapy, and previous studies have demonstrated that STE is feasible and convenient for early detection of cardiac dysfunction [13–15].

The aim of the present study was to identify a parameter for reliable detection of cardiac dysfunction via STE after chemotherapy in breast cancer patients. We hypothesed that longitudinal rotation (LR) motion could be detected in the hearts of breast cancer patients after chemotherapy, then tested the hypothesis by measuring the global peak systolic LR (PSLR) degrees in breast cancer patients after anthracycline-based chemotherapy. Cardiac dysfunction was then evaluated to confirm whether LR could serve as a new way to detect cardiac dysfunction after chemotherapy in breast cancer patients.

## RESULTS

### General characteristics of the patients

The general characteristics of the study group are listed in Table 1. The cumulative dose of epirubicin given was significantly higher than that of therarubicin.

**Table 1:** Baseline characteristics of the total population (mean±SD)

Variable	Baseline
Age (yrs)	49±8
Female	43(43)
Body surface area (m^2^)	1.65±0.12
Systolic blood pressure(mmHg)	121±11
Diastolic blood pressure (mmHg)	76±9
Heart rate (bpm)	77±3
Side of breast cancer	
Left	22(43)
Right	21(43)
Both	0(43)
Cardiovascular risk factors	
Diabetes	0(43)
Hypertension	0(43)
Hyperlipidemia	0(43)
Smoking history	0(43)
Cumulative dose of anthracycline (mg/m^2^)	
Epirubicin (27)	524±141* (95 % CI: 479-634)
Therarubicin (16)	336±115 (95 % CI: 275-398)
Chemotherapy	
FEC(5-FU, EPI/ THP, CTX)	30(43)
5-FU, EPI, CTX	17(30)
5-FU, THP, CTX	13 (30)
TEC(TXT, EPI/ THP, CTX)	13(43)
TXT, EPI, CTX	10 (13)
TXT, THP, CTX	3 (13)

5-FU: 5-flourouracil; EPI: epirubicin; THP: therarubicin; CTX: cyclophosphamide; TXT: taxotere.

The values at baseline compared with those at 3 weeks after the final cycle of chemotherapy, *P<0.05.

### Conventional two-dimensional echocardiographic parameters of the study groups

The conventional two-dimensional echocardio-graphic parameters of the study groups at the three time points are listed in Table 2. There were no significant differences among the study groups at the three separate time points, except that the e’_sep_ at baseline was higher than at 3 weeks and 6 months after the final cycle of chemotherapy. Although there were no significant differences among E, E/A, and e’_lat_ between the groups, the baseline values were higher than those at 3 weeks and 6 months after the final cycle of chemotherapy.

**Table 2:** Conventional two-dimensional echocardiographic parameters, speckle-tracking echocardiographic parameters and segmental and global peak systolic longitudinal rotation (PSLR) of the total population at baseline and 3 weeks and 6 months after the final cycle of chemotherapy (mean±SD)

Variable			Baseline	3 weeks	6 months	P-value
Conventional two-dimensional echocardiographic parameters		IVSd(mm)	8.93±0.99	8.67±0.78	8.72±0.80	0.342
		LVDd(mm)	44.98±3.03	45.63±2.95	45.95±3.07	0.314
		LVPWd(mm)	8.67±1.06	8.56±0.73	8.79±0.86	0.487
		LVSd(mm)	29.74±2.73	30.56±2.77	30.67±2.09	0.187
		LVEDV(ml)	61.44±13.77	64.35±13.38	64.37±13.48	0.516
		LVESV(ml)	22.23±6.41	23.12±7.57	23.42±6.79	0.723
		LVEF(%)	63.47±6.31	63.35±5.88	63.72±6.49	0.961
		E(m/s)	0.71±0.16	0.65±0.14	0.66±0.17	0.180
		A(m/s)	0.66±0.13	0.69±0.17	0.69±0.15	0.556
		E/A	1.13±0.39	0.99±0.32	1.01±0.36	0.144
		e’_Sep_	0.09±0.02*	0.07±0.02	0.08±0.02	**0.022**
		a’_Sep_	0.10±0.02	0.10±0.02	0.10±0.02	0.633
		e’_lat_	0.13±0.03	0.12±0.03	0.11±0.03	0.062
		a’_lat_	0.10±0.02	0.10±0.03	0.10±0.02	0.903
		E/e’_sep_	5.73±1.68	5.75±1.84	6.17±2.04	0.469
		E/e’_lat_	8.78±2.64	9.27±2.38	9.15±2.72	0.660
Speckle-tracking echocardiographic parameters	Peak velocity(cm/s)	Systolic	3.60±0.95	3.37±1.34	3.31±0.71	0.391
		Early-diastolic	-4.48±1.33*#	-3.97±1.15	-3.81±1.09	**0.028**
		Late-diastolic	-3.82±0.94	-3.78±0.83	-3.74±0.88	0.912
	Peak systolic strain(%)	Endomyocardium	-24.98±3.67	-23.73±6.32	-24.20±6.32	0.451
		Midmyocardium	-21.25±3.04	-20.76±2.48	-20.37±2.68	0.333
		epimyocardium	-18.59±2.61	-18.21±2.57	-17.63±2.28	0.206
	Peak strain rate(S^-1^)	Systolic	-1.30±0.21	-1.16±0.48	-1.21±0.17	0.139
		Early-diastolic	1.76±0.37	1.65±0.35	1.63±0.39	0.238
		Late-diastolic	1.19±0.30	1.45±1.67	1.17±0.30	0.335
	Peak systolic Displacement (mm)		9.79±1.82	8.72±5.06	9.21±1.53	0.308
PSLR	4-CH	Lateral wall	9.30±2.73*#	7.00±4.08	6.83±4.42	**0.005**
		Septal wall	-8.12±3.06	-8.87±2.90	-8.75±2.72	0.438
		Apex wall	1.51±3.34	-0.14±4.03	-0.14±3.78	0.064
		Global	-0.23±3.26*#	-1.91±3.27	-1.90±2.92	**0.020**

IVSd: interventricular septal thickness in end-diastolic period, LVDd: left ventricular diameter in end-diastolic period, LVPWd: left ventricular posterior wall thickness in end-diastolic period, LVSd: left ventricular diameter in end-diastolic period, LVEDV: left ventricular end-diastolic volume, LVESV: left ventricular end-systolic volume, LVEF: left ventricular ejection fraction, E: peak velocity during early diastole of anterior mitral leaflet, A: peak velocity during late diastole of anterior mitral leaflet, e’_Sep_: peak early diastolic annular velocities at septum positions using TDI, a’_Sep_: peak late diastolic annular velocities at septum positions using TDI, e’_lat_: peak early diastolic annular velocities at lateral positions using TDI, a’_lat_: peak late diastolic annular velocities at lateral positions using TDI. 4-CH: Apical 4- chamber view

The values at baseline compared with those at 3 weeks after the final cycle of chemotherapy, *P<0.05.

The values at baseline compared with those at 6 weeks after the final cycle of chemotherapy, #P<0.05.

“+” indicates counter-clockwise rotation and “-” indicates clockwise rotation.

### STE parameters

**Figure 1:**
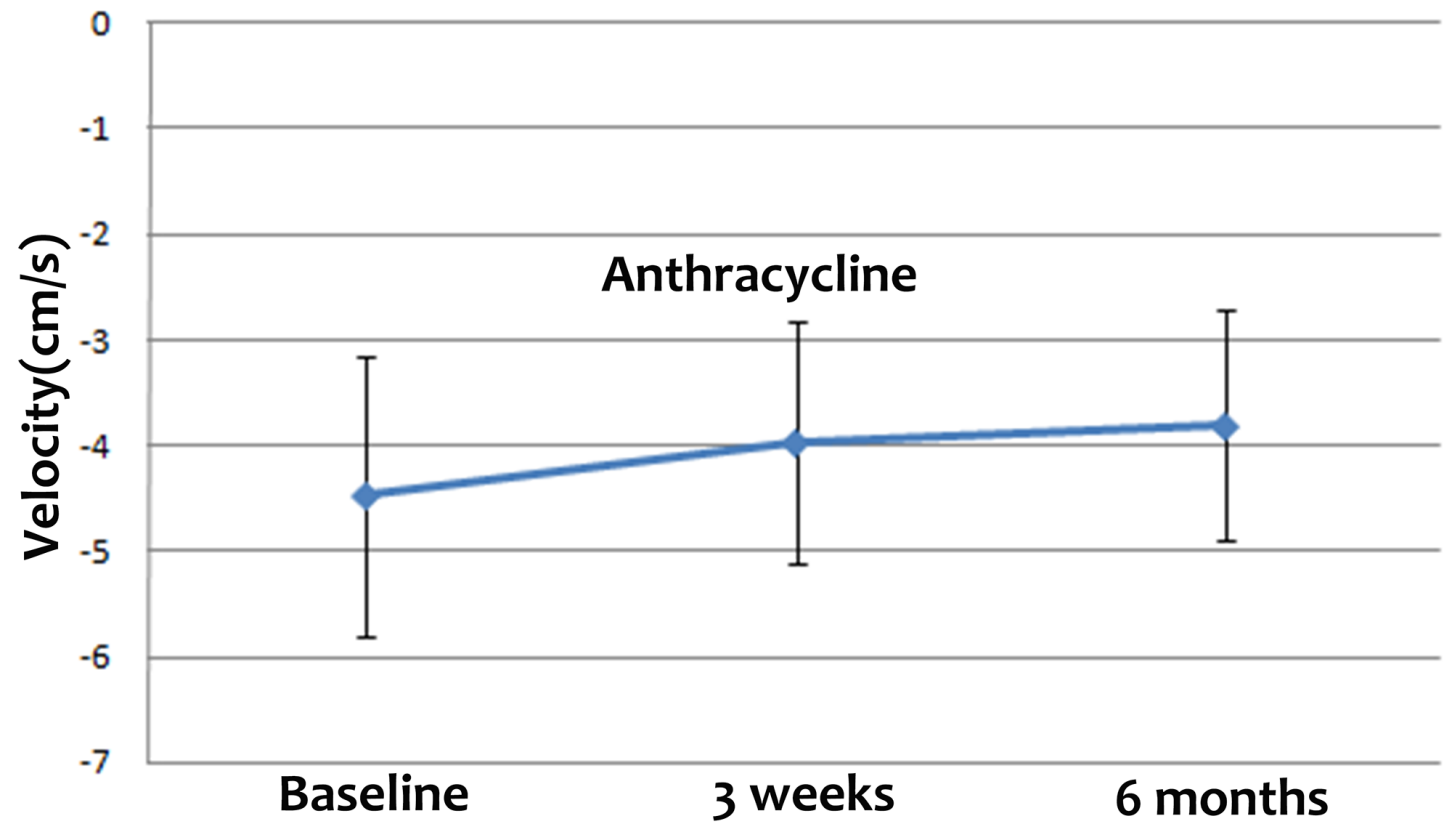
Peak early-diastole LV wall velocities at baseline and 3 weeks and 6 months after the final cycle of chemotherapy The peak early-diastole LV wall velocity at baseline was significantly higher than at 3 weeks and 6 months after the final cycle of chemotherapy.

**Figure 2:**
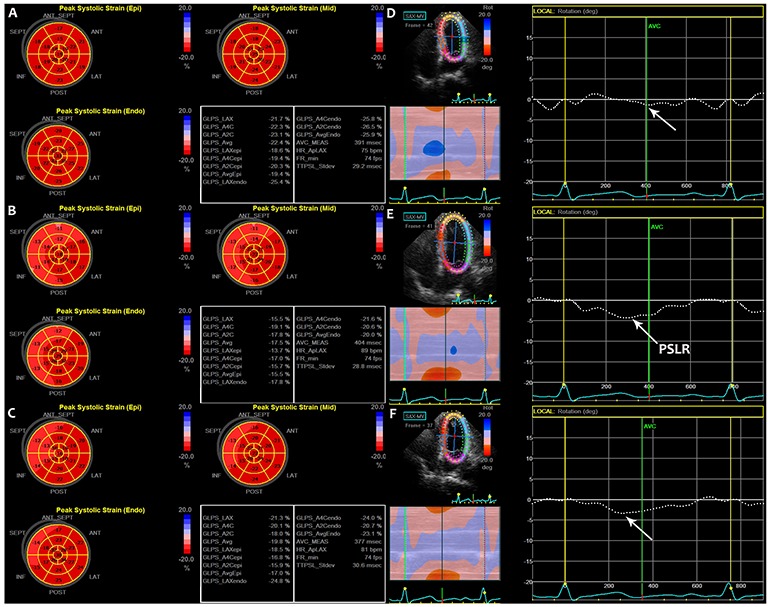
Bulls eye diagrams of the peak systolic strain of LV in the subendocardial, midmyocardial, and subepicardial layers at three time points **(A, B, C)**. (A) At baseline, (B) 3 weeks after the final cycle of chemotherapy, and (C) 6 months after the final cycle of chemotherapy. Global PSLR in breast cancer patients at three separate time points **(D, E, F)**. (D) At baseline, (E) 3 weeks after the final cycle of chemotherapy, and (F) 6 months after the final cycle of chemotherapy. PSLR: peak systolic longitudinal rotation.

The STE parameters of the study groups at the three time points are listed in Table 2. The peak early-diastole LV wall velocity at baseline was significantly higher than at 3 weeks and 6 months after the final cycle of chemotherapy (Figure 1). Although there were no significant differences among the peak systolic longitudinal strains of the different myocardial layers, the values at baseline were higher than those at 3 weeks and 6 months after the final cycle of chemotherapy (Figure 2A, 2B and 2C). There were no differences among the peak longitudinal strain rates and displacements.

### Segmental and global PSLR

**Figure 3:**
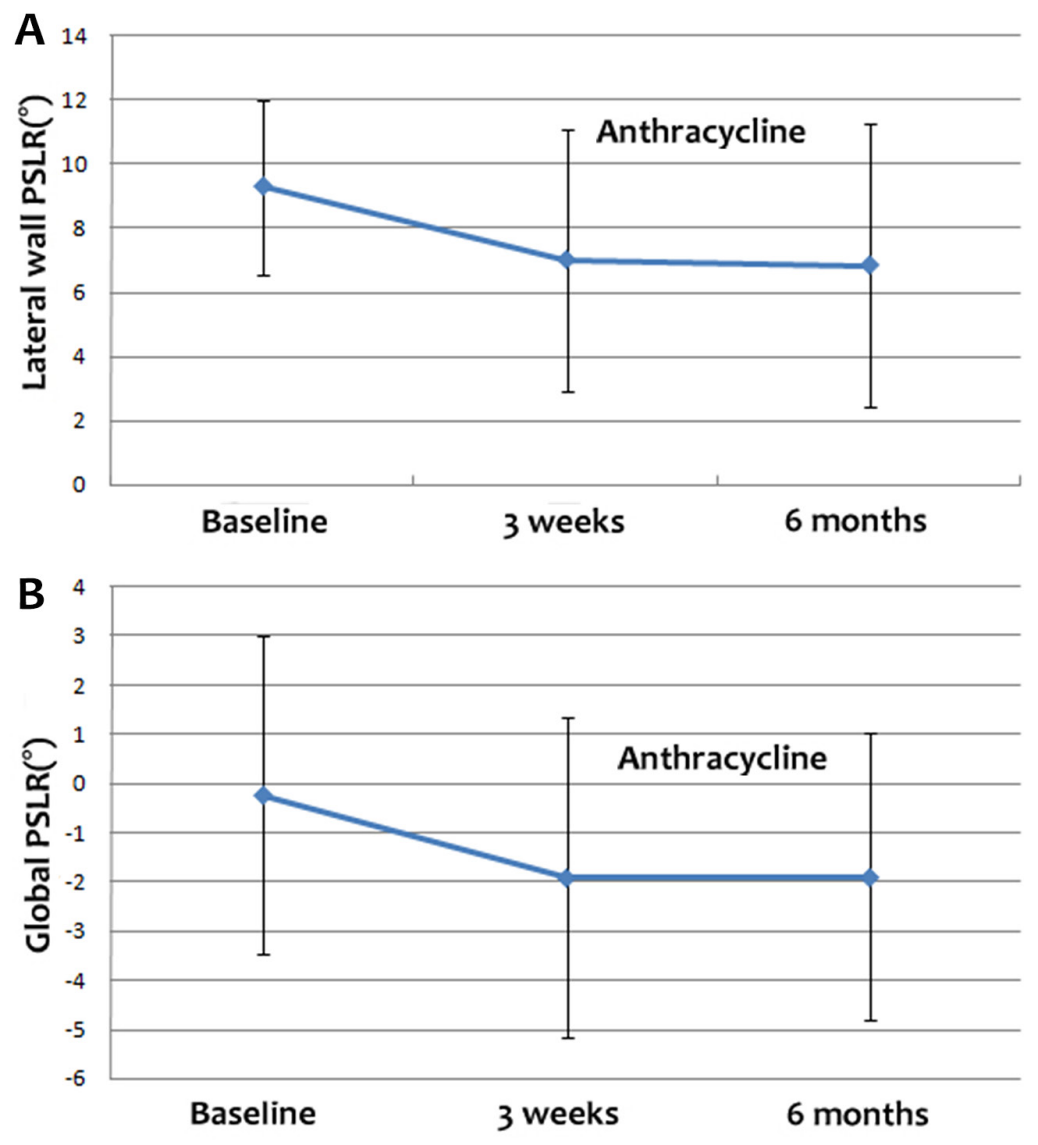
Lateral wall **(A)** and global PSLR **(B)** at baseline and 3 weeks and 6 months after the final cycle of chemotherapy. The absolute value of the lateral wall PSLR was higher at baseline than at 3 weeks and 6 months after the final cycle of chemotherapy, while the absolute value of the global PSLR at baseline was lower than at 3 weeks and 6 months after the final cycle of chemotherapy.

The segmental and global PSLR values of the study groups at the three time points are shown in Table 2. In the apical 4-chamber view, the lateral wall rotated counter-clockwise, whereas the septal wall rotated clockwise. The global PSLR at baseline was small, and significant differences were detected between the lateral wall and global PSLR values at baseline and those at 3 weeks and 6 months after the final cycle of chemotherapy (Figure 2D, 2E and 2F). The absolute value of the lateral wall PSLR at baseline was higher than the values at 3 weeks and 6 months after the final cycle of chemotherapy (Figure 3A), whereas the absolute value of the global PSLR at baseline was lower than the values at 3 weeks and 6 months after the final cycle of chemotherapy (Figure 3B). The values of the lateral wall and global PSLR did not change significantly from 3 weeks to 6 months after the final cycle of chemotherapy.

### Correlations between the global PSLR and LVEF, E, A, E/A, e’_sep_, a’_sep_, e’_lat_, a’_lat_, E/ e’_sep_, E/ e’_lat_ and cumulative dose of anthracycline

**Figure 4:**
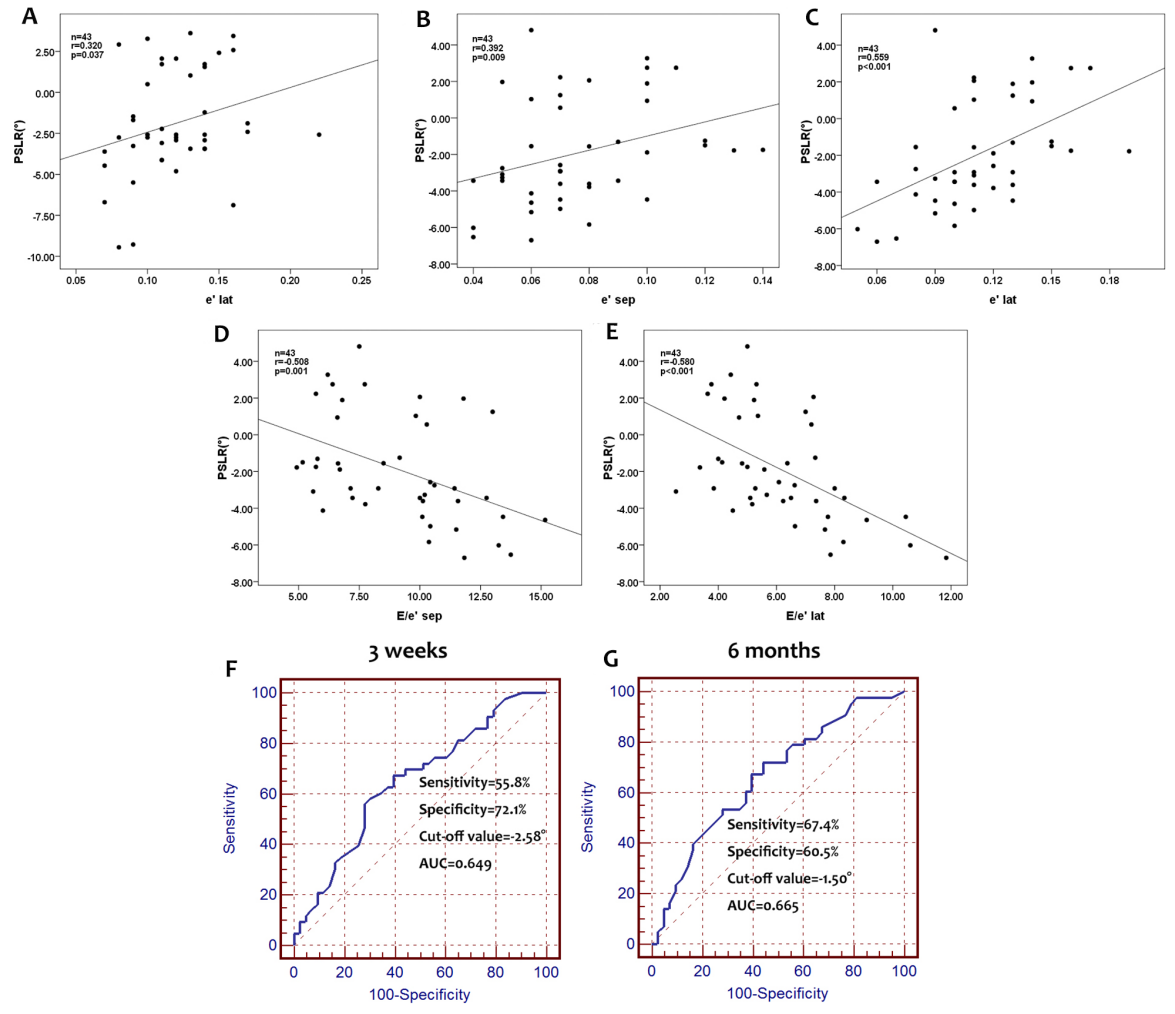
Correlation between global PSLR and e’_lat_ at 3 weeks after the final cycle of chemotherapy Global PSLR positively correlated with the e’_lat_ at 3 weeks, and thus, breast cancer patients with a higher e’_lat_ at 3 weeks after the final chemotherapy cycle may have a higher global PSLR **(A)**. Correlations between global PSLR and e’_sep_, e’_lat_, E/e’_sep_, and E/e’_lat_ at 6 months after the final cycle of chemotherapy. Global PSLR positively correlated with e’_sep_ and e’_lat_ and negatively correlated with E/e’_sep_ and E/e’_lat_. Breast patients with a higher e’_sep_ and e’_lat_, and lower E/e’_sep_ and E/e’_lat_ may have a higher global PSLR **(B, C, D, E)**. ROC analysis for determining the accuracy of PSLR for identifying cardiac dysfunction in breast cancer patients. The sensitivity and specificity for a PSLR cut-off value of -2.58° at 3 weeks after the final cycle of chemotherapy were 55.8% and 72.1%, respectively, with an area under the ROC curve of 0.649 **(F)**. At 6 months after the final cycle of chemotherapy, these values were 67.4% and 60.5% for a PSLR, cut-off value of -1.50°, with an area under the ROC curve of 0.665 **(G)**.

The results of correlation tests are listed in Table 3. At baseline, no correlation was found between the global PSLR and LVEF, E, A, E/A, e’_sep_, a’_sep_, e’_lat_, a’_lat_, E/ e’_sep_ and E/ e’_lat_.However, at 3 weeks after the final cycle of chemotherapy, the global PSLR was positively correlated with the e’_lat_. Breast cancer patients with a higher e’_lat_ were more likely to have a higher global PSLR (Figure 4A). At 6 months after the final cycle of chemotherapy, the global PSLR was positively correlated with e’_sep_ and e’_lat_, and negatively correlated with E/e’_sep_ and E/e’_lat_. Breast cancer patients with a higher e’_sep_ and e’_lat_ and lower E/e’_sep_ and E/e’_lat_ were more likely to have a higher global PSLR (Figure 4B, 4C, 4D and 4E).

**Table 3:** Correlations between global PSLR and 2D, Doppler values and cumulative dose of anthracycline.

	Global PSLR					
	Baseline		3 weeks		6 months	
	r	p	r	p	r	p
LVEF	0.075	0.634	-0.211	0.175	-0.233	0.133
E	-0.01	0.950	-0.038	0.811	-0.110	0.481
A	-0.214	0.169	-0.046	0.770	-0.273	0.076
E/A	0.155	0.322	0.044	0.779	0.078	0.617
e’_Sep_	0.261	0.091	0.140	0.372	0.392	**0.009**
a’_Sep_	-0.116	0.458	-0.258	0.095	-0.202	0.193
e’_lat_	0.291	0.058	0.320	**0.037**	0.559	**<0.001**
a’_lat_	0.094	0.548	-0.099	0.529	0.014	0.929
E/e’_sep_	-0.272	0.078	-0.169	0.280	-0.508	**0.001**
E/e’_lat_	-0.202	0.194	-0.258	0.070	-0.580	**<0.001**
Cumulative dose			-0.180	0.248	-0.147	0.347

### ROC analysis for detecting the accuracy of PSLR for detecting cardiotoxicity in breast cancer patients

The areas under ROC curves (AUCs) were measured to determine the cut-off values for the global PSLR with the optimal sensitivity, specificity, and accuracy for assessing cardiotoxicity with regard to LV function. The AUC values at 3 weeks and 6 months after the final cycle of chemotherapy in breast cancer patients were 0.649 and 0.665, respectively. The optimal cut-off values for global PSLR at 3 weeks and 6 months after the final cycle of chemotherapy were -2.58° and -1.50°, respectively. The sensitivity and specificity for the global PSLR at 3 weeks after the final cycle of chemotherapy were 55.8% and 72.1%, respectively, and the corresponding values at 6 months after the final cycle of chemotherapy were 67.4% and 60.5%, respectively (Figure 4F and 4G).

### Differing effects of epirubicin-based and therarubicin-based chemotherapy

**Figure 5:**
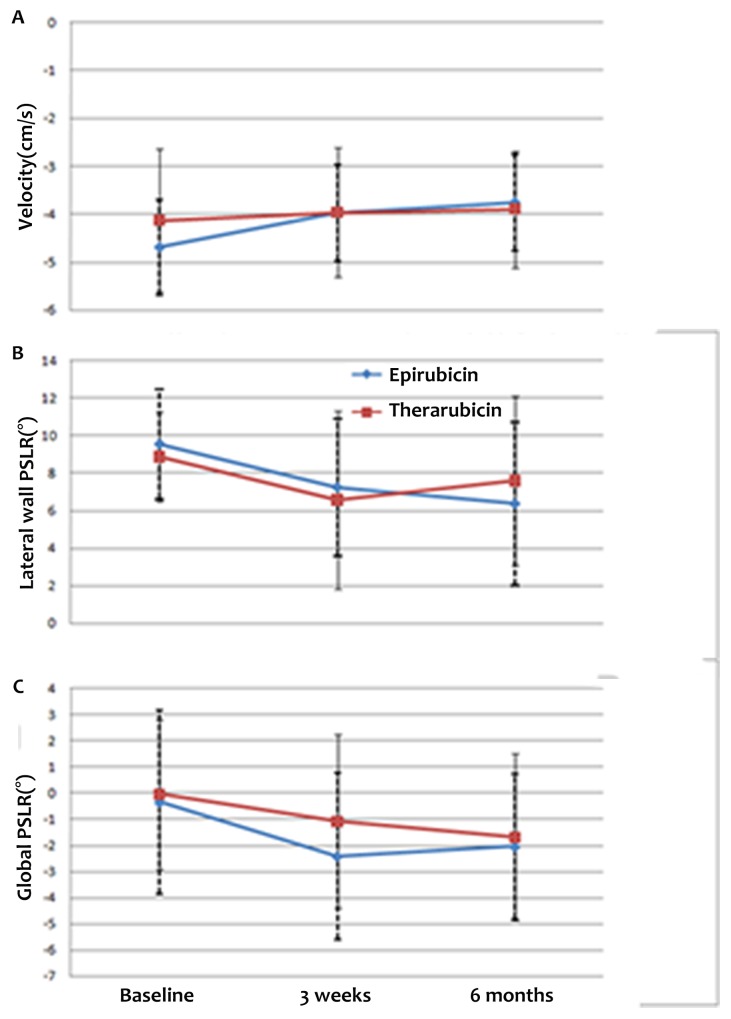
Differences in peak early-diastole LV wall velocity **(A)** and lateral wall **(B)** and global PSLR **(C)** after epirubicin-based chemotherapy versus therarubicin-based chemotherapy at baseline and 3 weeks and 6 months after the final cycle of chemotherapy. The absolute values of the peak early-diastole LV wall velocity and the lateral wall PSLR at baseline were higher than those at 3 weeks and 6 months after the final cycle of chemotherapy, whereas the absolute value of global PSLR at baseline was lower than at 3 weeks and 6 months after the final cycle of chemotherapy in both epirubicin-based and therarubicin-based chemotherapy groups.

The STE parameters of patients who received epirubicin-based chemotherapy are compared to those of patients who received therarubicin-based chemotherapy in Table 4. In the group that received epirubicin-based chemotherapy, the peak early-diastole LV wall velocity, and lateral wall and global PSLR differed significantly among the three time points; however, these values did not change significantly over time after therarubicin-based chemotherapy. The absolute value of the peak early-diastole LV wall velocity and the lateral wall PSLR at baseline were higher than those at 3 weeks and 6 months after the final cycle of chemotherapy, whereas the absolute value of global PSLR at baseline were lower than the values at 3 weeks and 6 months after the final cycle of chemotherapy in both the epirubicin-based and therarubicin-based chemotherapy groups. The values of these parameters did not differ significantly between 3 weeks and 6 months after the final cycle of chemotherapy (Figure 5A, 5B and 5C).

**Table 4:** Detection of differences in epirubicin-based cardiotoxicity and therarubicin-based cardiotoxicity (mean±SD)

Variable	Epirubicin				Therarubicin			
	Baseline	3 weeks	6 months	P	Baseline	3 weeks	6 months	P
Velocity (peak early-diastolic) (cm/s)	-4.69±1.22*#	-3.97±1.05	-3.76±1.02	**0.007**	-4.13±1.49	-3.97±1.35	-3.90±1.22	0.128
Lateral wall PSLR (°)	9.55±2.93*#	7.25±3.67	6.37±4.38	**0.007**	8.87±2.39	6.58±4.78	7.60±4.53	0.286
Global PSLR (°)	-0.34±3.51*	-2.41±3.18	-2.04±2.80	**0.044**	-0.04±2.88	-1.07±3.33	-1.67±3.19	0.344

### Reproducibility and repeatability

The results for the intraobserver and interobserver variabilities for the global PSLR upon repeated measurements in all study patients are shown in Table 5.

**Table 5:** Interobserver and intraobserver reproducibility and repeatability

Variable	Global PSLR(°)			
	Interobserver		Intraobserver	
	Mean± SD	95 % CI	Mean ±SD	95 % CI
Baseline	-0.19±0.49	-1.18∼0.80	-0.19±0.48	-1.16∼0.78
3 weeks	-1.99±0.51	-3.02∼-0.96	-1.87±0.50	-2.89∼ -0.86
6 months	-1.96±0.45	-2.86∼-1.05	-1.90±0.44	-2.79∼-1.01

## DISCUSSION

As is well known, chemotherapy can reduce the recurrence of breast cancer, especially when used in combination with anthracyclines. However, the cardiotoxicity of anthracyclines is becoming a major concern. Early detection of cardiotoxicity is becoming increasingly important. The main findings of this study were: ① cardiac diastolic and systolic dysfunction was found in breast cancer patients after anthracycline-based chemotherapy; ② LR motion is a new parameter for detecting cardiotoxicity at 3 weeks and 6 months after the final cycle of chemotherapy; and ③ subgroup analysis indicated that the cardiotoxicity of epirubicin-based chemotherapy maybe stronger than that of therarubicin-based chemotherapy.

In clinical practice, LVEF is quantified as a measure of cardiac function. In the present study, we found that there were no significant differences in LVEF among the three time points. Thus, LVEF cannot be used in clinical practice for early detection of cardiotoxicity. With its angle independency and high reproducibility, STE is widely used for detection of cardiac function [16–18]. Ho et al [19] used STE to examine the long-term effects of standard chemotherapy on myocardial function in asymptomatic breast cancer survivors and found that the E/A ratio, global E’, and global longitudinal 2D strain were reduced in comparison with those of controls. Fallah-Rad et al [3] used cardiac biomarkers, tissue velocity (TVI), strain imaging, and CMRI to predict early LV dysfunction in breast cancer patients treated with adjuvant trastuzumab therapy. They found that the peak global longitudinal and radial strain decreased early in the group with trastuzumab-mediated cardiotoxicity. Portugal et al [16] used speckle tracking imaging to determine the relationship between global and regional longitudinal strain (GLS) and chemotherapy-induced cardiotoxicity in breast cancer patients and concluded that impaired GLS in patients with preserved LVEF was independently associated with an increased incidence of chemotherapy-induced cardiotoxicity. Our results were consistent with those of this previous research. Cardiac diastolic and systolic dysfunction was found after administration of anthracycline-based chemotherapy, based on lower values of e’_Sep_, peak early-diastolic LV wall velocity, and longitudinal strain in different myocardial layers at 3 weeks and 6 months after the final cycle of chemotherapy in comparison with the baseline values. The reasons for these results may be related to LV wall stress and local differences in the activation of signaling pathways involved in apoptosis or fibrosis.

LR, was first reported by Popović [20], represents the rotational motion in the long axis of the heart. In ischemic cardiomyopathy, dilated cardiomyopathy, and hypertension patients, as well as in acute myocardial ischemia animals, LR motion in the heart has been reported [21–23]. A normal myocardial fiber consists of longitudinal and circumferential fibers. Subendocardial and subepicardial myocardium are mainly composed of longitudinal fibers, and the middle layer is composed of circumferential fibers [24]. During systole and diastole, two motions are produced: short and long axis motions. In systole, when viewed from the apex, the apex rotates counter-clockwise, while the base rotates clockwise. This twisting motion of the left ventricle can be described as ‘‘the wringing of a linen cloth to squeeze out water’’ [15]. When viewed from its long axis, in systole, shortening of the long axis and thickening of the walls can be observed.

Previous studies indicated that the degree of PSLR in a normal heart is small, and our results agree with this [21–23]. The global PSLR at baseline in our study was -0.23±3.26°. At 3 weeks and 6 months after the final cycle of chemotherapy, the absolute values were higher than that at baseline. However, the value of the lateral wall PSLR at baseline was lower than those at 3 weeks and 6 months after the final cycle of chemotherapy. These results may demonstrate systolic dysfunction after anthracycline-based chemotherapy. The reason for such LR may be related to changes in the fiber arrangement by 3 weeks and 6 months after the final cycle of chemotherapy. Anthracyclines induce the formation of reactive oxygen species, which in excess can result in DNA and cytosolic damage, with subsequent loss of cellular integrity and apoptosis, inducing disarray and imbalance of the myocardial fibers [25].

In the present study, no correlations were found between the baseline values of global PSLR and LVEF, E, A, E/A, e’_sep_, a’_sep_, e’_lat_, a’_lat_, E/e’_sep_, E/e’_lat_ and cumulative dose of anthracycline. At 3 weeks after the final cycle of chemotherapy, breast patients with a higher e’_lat_ were more likely to have a higher global PSLR. At 6 months after the final cycle of chemotherapy, breast patients with higher e’_sep_ and e’_lat_ and lower E/e’_sep_ and E/e’_lat_ values were more likely to have a higher global PSLR. These findings indicated that although the LV filling pressure did not differ among the three time points, a lower LV filling pressure may correspond to a higher global PSLR.

The AUC values for PSLR at 3 weeks and 6 months after the final cycle of chemotherapy in breast cancer patients were 0.649 and 0.665, respectively. The sensitivity and specificity values for a PSLR cut-off value of -2.58° at 3 weeks after the final cycle of chemotherapy were 55.8% and 72.1%, respectively, and those for a PSLR cut-off value of -1.50° at 6 months after the final cycle of chemotherapy were 67.4% and 60.5%, respectively. From these results, we found that use of the global PSLR to detect cardiotoxicity after anthracycline-based chemotherapy was reliable.

When we compared parameters of cardiac function between the patients who received epirubicin-based and therarubicin-based chemotherapy, we found that after epirubicin-based chemotherapy, the peak early-diastole LV wall velocity and lateral wall and global PSLR differed significantly among the three time points. However, no differences between the time points were observed in patients who received therarubicin-based chemotherapy. The absolute values of peak early-diastole LV wall velocity and the lateral wall PSLR at baseline were higher than those at 3 weeks and 6 months after the final cycle of chemotherapy, whereas the absolute value of global PSLR at baseline was lower than those at 3 weeks and 6 months after the final cycle of chemotherapy, in both the epirubicin-based and therarubicin-based chemotherapy groups. From this, we found that the cardiotoxicity of epirubicin-based chemotherapy may be stronger than that of therarubicin-based chemotherapy.

Finally, the lack of significant differences in the values of cardiac function parameters between 3 weeks and 6 months after the final cycle of chemotherapy illustrated that the anthracycline-induced cardiotoxicity is irreversible. But to state this surely, more breast cancer patients should be increased and also it needs longer follow up.

## MATERIALS AND METHODS

### Study sample

This study was approved by the ethics committee of ChangZhou No. 2 People’s Hospital, affiliated to NanJing Medical University. All patients provided consent for participation in the study.

Forty-three female breast cancer patients were enrolled. The inclusion criteria were: ① treatment with epirubicin or therarubicin; and ② LVEF >50%. None of the patients had a history of cardiovascular disease, hypertension, or diabetes mellitus, all of them were asymptomatic before the chemotherapy; and ③ All enrolled breast cancer patients did not perform radiotherapy.

In total, the patients were evaluated at three separate time points: ① baseline: 1–3 days before the initiation of anthracycline-based chemotherapy; ② 3 weeks after the final cycle of chemotherapy; and ③ 6 months after the final cycle of chemotherapy.

### Conventional 2D echocardiography

At each visit, all patients underwent conventional 2D echocardiography (Vivid E9, GE Healthcare). Interventricular septum thickness at end-diastole (IVSd), LV posterior wall thickness in end-diastole (LVPWd), and LV diameter at end-diastole (LVDd) and end-systole (LVSd) were measured in the parasternal long axis view of the LV by M-mode. Biplane Simpson’s method was used to measure the LV end-diastolic and systolic volume (LVEDV and LVESV) for calculation of the LVEF. The peak early and late diastolic mitral annular velocities (E and A, respectively) were measured by pulsed-wave Doppler, and the ratio of E/A was calculated. The peak early (e’) and late (a’) diastolic annular velocities were obtained at the septum (e’_sep_) and lateral (e’_lat_) positions using TDI, and E/ e’_sep_ and E/ e’_lat_ were measured.

In each group, ECG leads were connected to the patients. For offline analysis, the standard high frame rate (>36 /s) of the apical 3-, 4-, and 2-chamber views of three consecutive cycles were measured while patients held their breath.

### Analysis of LV function

The apical 3-, 4-, and 2- chamber views were analyzed using 2D-STE software (2D-Strain, EchoPac PC 113, GE Healthcare, Horten, Norway) by one experienced cardiologist. The peak systolic and diastolic longitudinal velocities and strain rates as well as the peak systolic longitudinal strain of three different layers and displacement of LV were measured.

We defined LR as the global rotation of the LV cross section. Using the SAX-MV option of the Echopac in the apical 4-chamber view, the subendocardial layer was displayed. The software automatically created a region of interest (ROI) that included the subendocardial, midmyocardial, and subepicardial layers. The LV region was divided into five segments: base-septal, middle-septal, apex, middle-lateral, and base-lateral. The segmental and global PSLR of the LV was assessed via the software.

### Statistical analysis

All data analyses were performed using SPSS 17.0 software (SPSS, Chicago, IL, USA). Data are presented as the mean ± standard deviation (SD). Any difference was considered statistically significant in all tests when the P-value was <0.05. Kolmogorov-Smirnov’s test was used to detect the normality of all the values. Differences among the values at the three separate time points were compared with one-way analysis of variance. Comparisons of two samples were made using the Student–Newman–Keuls test. We defined the global PSLR at the baseline as the normal state and considered the values at 3 weeks and 6 months after the final cycle of chemotherapy as the test values. The values for global PSLR of the heart in breast cancer patients were determined from receiver operating characteristic (ROC) curve analysis. Yoden’s index was used to determine the cut-off point with the best composite of specificity and sensitivity. Correlations between the global PSLR and LVEF, E, A, E/A, e’_sep_, a’_sep_, e’_lat_, a’_lat_, E/ e’_sep_, E/ e’_lat_, and cumulative dose of anthracycline were determined by Spearman’s correlation.

### Reproducibility and repeatability

Intraobserver and interobserver variability for global PSLR were determined by repeating measurements in all enrolled breast cancer patients. For the second intraobserver measurements, the observer was ‘‘blinded’’ to results of the initial measurements.

## CONCLUSIONS

In the present study, we found that patients experienced cardiac diastolic and systolic dysfunction after anthracycline-based chemotherapy, and the cardiotoxicity of epirubicin-based chemotherapy may be stronger than that of therarubicin-based chemotherapy. The PSLR represents a new parameter that can be used for detection of cardiotoxicity in breast cancer patients after anthracycline-based chemotherapy.

## Abbreviations

LV: Left ventricular
LVEF: Left ventricular ejection fraction
TDI: Tissue Doppler imaging
STE: Speckle tracking echocardiography
CMRI: Cardiac magnetic resonance imaging
LR: Longitudinal rotation
PSLR: Peak systolic longitudinal rotation
GLS: Global longitudinal strain
IVSd: Interventricular septum thickness at end-diastole
LVPWd: Left ventricular posterior wall thickness at end-diastole
LVDd: Left ventricular diameter at end-diastole
LVSd: Left ventricular diameter at end-systole
LVEDV: Left ventricular end-diastolic volume
LVESV: Left ventricular end-systolic volume
